# Distinct Immune Profiles of Exhausted Effector and Memory CD8^+^ T Cells in Individuals With Filarial Lymphedema

**DOI:** 10.3389/fcimb.2021.680832

**Published:** 2021-08-11

**Authors:** Sacha Horn, Dennis Borrero-Wolff, Manuel Ritter, Kathrin Arndts, Anna Wiszniewsky, Linda Batsa Debrah, Alexander Y. Debrah, Jubin Osei-Mensah, Mkunde Chachage, Achim Hoerauf, Inge Kroidl, Laura E. Layland

**Affiliations:** ^1^Division of Infectious Diseases and Tropical Medicine, University Hospital Munich, Ludwig-Maximilians-Universität (LMU), Munich, Germany; ^2^Institute for Medical Microbiology, Immunology and Parasitology (IMMIP), University Hospital Bonn, Bonn, Germany; ^3^German-West African Centre for Global Health and Pandemic Prevention (G-WAC), Partner Site, Bonn, Bonn, Germany; ^4^Kumasi Centre for Collaborative Research in Tropical Medicine (KCCR), Filariasis Unit, Kumasi, Ghana; ^5^Department of Clinical Microbiology, School of Medicine and Dentistry, Kwame Nkrumah University of Sciences and Technology, Kumasi, Ghana; ^6^German-West African Centre for Global Health and Pandemic Prevention (G-WAC), Partner Site, Kumasi, Kumasi, Ghana; ^7^Faculty of Allied Health Sciences, Kwame Nkrumah University of Sciences and Technology, Kumasi, Ghana; ^8^National Institute for Medical Research (NIMR)-Mbeya Medical Research Center (MMRC), Department of Immunology, Mbeya, Tanzania; ^9^University of Dar es Salaam-Mbeya College of Health and Allied Sciences (UDSM-MCHAS), Department of Microbiology and Immunology, Mbeya, Tanzania; ^10^German Centre for Infection Research (DZIF), Neglected Tropical Disease, partner site, Bonn-Cologne, Bonn, Germany; ^11^German Centre for Infection Research (DZIF), Neglected Tropical Disease, partner site, Munich, Munich, Germany

**Keywords:** Filariae, CD8^+^ T cell exhaustion, *Wuchereria bancrofti* infection, lymphatic filariasis, immune modulation, lymphedema, memory and effector T cell subsets

## Abstract

CD8^+^ T cells are crucial for the clearance of viral infections, and current research begins to highlight their importance in parasitic diseases too. In-depth research about characteristics of CD8^+^ T-cell subsets and exhaustion remains uncertain, especially during filariasis, a chronic helminth infection. Lymphatic filariasis, elicited by *Wuchereria bancrofti*, remains a serious health problem in endemic areas in Ghana, especially in those suffering from morbidity due to lymphedema (LE). In this observational study, the characteristics and profiles of CD8^+^ T cells were compared between asymptomatic *Wuchereria bancrofti*-infected individuals, uninfected endemic normals, and those with LE (grades 2–6). Focusing on exhausted memory (CD8^+^ex_mem_: CD8^+^ T-bet^dim^Eomes^hi^) and effector (CD8^+^ex_eff_: CD8^+^T-bet^hi^Eomes^dim^) CD8^+^ T-cell subsets, advanced flow cytometry revealed that LE individuals presented reduced frequencies of IFN-γ^+^CD8^+^ex_mem_ T cells expressing Tim-3 or LAG-3 which negatively correlated to the presence of LE. Moreover, the LE cohort further showed significantly higher frequencies of IL-10^+^CD8^+^ex_eff_ T cells expressing either Tim-3, LAG-3, CD39, KLRG-1, or PD-1, all associated markers of exhaustion, and that these frequencies positively correlated with the presence of LE. In summary, this study shows that distinct exhausted CD8^+^ T-cell subsets are prominent in individuals suffering from LE, suggesting that enhanced inflammation and constant immune activation might drive exhaustion of CD8^+^ T cells. Since T-cell exhaustion is known to be associated with insufficient control of persisting antigen, the data presented here reveals that these CD8^+^ T-cell exhaustion patterns in filarial LE should be taken into consideration for prevention and control management of LE.

## Introduction

In filarial endemic areas, chronic helminth infections remain a substantial public health burden leading to considerable disease morbidity and mortality (Hotez et al., 2008). Efficient control of chronic helminth infections requires the interplay of multiple adaptive cell types including antibody-secreting B-cell and T-cell populations (Adjobimey and Hoerauf, 2010; Ludwig-Portugall and Layland, 2012; Zander and Butler, 2013). The prerequisite of CD8^+^ T cells for the recognition and elimination of intracellular pathogens is well documented, but there remains a paucity about the role of CD8^+^ T cells in chronic helminth infections such as lymphatic filariasis (Bannard et al., 2009). Moreover, little is known about the presence or role of exhausted CD8^+^ T-cell subsets developing in chronic helminth infections; these exhausted CD8^+^ T-cell profiles are known to be distinct from memory or acutely activated populations (Wherry and Kurachi, 2015). Decreased functional responses by exhausted CD8^+^ T cells occur in a step-wise fashion: first, there is loss of IL-2 secretion, followed by cells reducing their proliferative capacity and finally, cytotoxic function (Zander and Butler, 2013). Loss of TNF-α and IFN-γ secretion also occurs in severely exhausted cells, and these effects are usually linked to deficiencies in pathogen control (Wherry et al., 2003; Wherry, 2011). A clear understanding of how CD8^+^ T cells function is one of the key factors when developing immunotherapeutic strategies. For a better characterization of exhausted CD8^+^ T-cell phenotypes, Buggert and colleagues were able to link the expression levels of T-box transcription factors, T-bet and Eomesdermin (Eomes), to exhausted CD8^+^ T cells (Buggert et al., 2014). Furthermore, expression levels of programmed cell death-1 (PD-1), lymphocyte activation gene 3 (LAG-3), killer cell lectin-like receptor subfamily G member 1 (KLRG-1), CD39, T-cell immunoglobulin and mucin-domain containing-3 (Tim-3), and IL-10 have been associated with an exhausted phenotype, and some of these markers have been associated with adaptive immunity during helminth infections (Layland et al., 2010; Zander and Butler, 2013; Wherry and Kurachi, 2015; Taylor et al., 2017; Lima et al., 2018).

Worldwide, approximately 65–68 million individuals are infected with lymphatic filariasis (Ramaiah and Ottesen, 2014; GBD, 2018) with about 40 million seriously incapacitated and disfigured with hydrocele or lymphedema (LE), making it a major public health concern (Ramaiah and Ottesen, 2014; Clayton et al., 2015; WHO, 2020). In general, LE is subdivided into seven stages depending on swelling (reversible or non-reversible), leg volume, and presence of skin folds, nodules, knobs, and lesions (Dreyer et al., 1999; WHO, 2001). More than 90% of individuals suffering from LF are infected with *Wuchereria bancrofti (W. bancrofti)*, especially in Sub-Saharan Africa (Ottesen, 2006). Chronic infections with *W. bancrofti* can develop into either hydrocele or LE, but the majority of patients maintain asymptomatic infections (Simonsen et al., 2014). Established asymptomatic infections with *W. bancrofti* are associated with systemic activation of CD4^+^ T cells, decreased Th2 response, a regulatory milieu with dominant IgG4 and IL-10, and the presence of regulatory T- and B-cell populations (Arndts et al., 2012; Babu and Nutman, 2014; Kroidl et al., 2019; Ritter et al., 2019). On the other hand, LE is associated with constant immune activation, increased pro-inflammatory responses, and failure of antigen-specific induction of T-cell hypo-responsiveness (Babu and Nutman, 2012; Babu and Nutman, 2014). Interestingly, CD8^+^ T cells and IL-26-expressing CD8^+^ T cells were predominately found in biopsies and peripheral blood of LE patients, respectively (Freedman et al., 1995; Anuradha et al., 2014a). Expanding on these initial findings, this study focuses on identifying characteristics of CD8^+^ T cells in individuals living in endemic areas of *W. bancrofti* and revealed distinct exhausted memory and effector CD8^+^ T-cell populations in patients with clinical pathology.

## Materials and Methods

### Ethics

In 2018, samples were taken from participants from the Upper East Region of Ghana (Navrongo, Kassena-Nankana Municipal District) as part of a study funded by the German Research Council (RHINO). Participants over the age of 18 years were enrolled, and written informed consent was obtained from all participants before sample collection. Ethical clearance was given by the Committee on Human Research Publication and Ethics at the Kwame Nkrumah University of Science and Technology in Kumasi, Ghana (CHRPE/AP/144/20), the Ethics Committee at the University Hospital of Bonn, Germany (Lfd. Nr. 041/18), and the Ethics Committee of the LMU Munich, Germany (18-377).

### Study Population and Parasitic Assessment

For flow cytometry analysis, blood samples were procured from a group of uninfected endemic normals (EN, n = 58), *Wuchereria bancrofti*-infected (Wb-infected, n = 29), or those individuals presenting LE (n = 37). In order to obtain a more comprehensive overview of the cohort, an epidemiological-based survey was conducted which included questions about age, gender, the years they lived in the endemic area, and the number of times they had received Ivermectin (IVM) and Albendazole (ALB) as part of mass drug administration programs (Card et al., 2012). All Wb-infected individuals tested positive with the Filariasis Test Strip (FTS; previously Alere, now Abbott Laboratories, Chicago, USA) and the TropBio Og4C3 Filariasis Antigen ELISA (Cellabs, Brookvale, Australia) and had no signs of pathology related to LF. The EN cohort was negative for both tests, had no signs of pathology related to LF, and had been living in the endemic area for at least 5 years. The LE cohort was defined according to the Dreyer staging protocol (Dreyer et al., 2003). All participants were found to be in general good health with no clinical visible or known other infections. All other infections are assumed to be equally distributed between the three groups. **Table 1** shows the descriptive statistics for the cohort.

**Table 1:** Study population characteristics.

	EN	Wb-inf.	LE
Sample size (n)	58	29	37
Mean age [range]	45.1 [21–81]	44.2 [20–83]	48.4 [26–64]
Gender (Female : Male) [%]	39:19 [67:33]	17:12 [59:41]	30:7 [81:19]
Mean years living in the endemic area [range]	39.2 [6–81]	42.8 [20–83]	48.4 [26–64]
Median MDA rounds [range]	2 [0–6]	4 [1–8]	6 [1–15]
Median lymphedema stage [SD]	NA	NA	3 [1.7]
FTS result/TropBio result	-/-	+/+	-/-*

*Data based on n = 27 PBMC LE samples which were used for expansive exhausted CD8^+^ T-cell panel.

Study participants were characterized as either endemic normal (EN), Wuchereria bancrofti infected (Wb-inf.), or with lymphedema pathology (LE). EN participants were negative for both Filariasis Test Strip (FTS) and TropBio Og4C3 Filariasis Antigen ELISA (TropBio), Wb-infected participants were positive for both the FTS and TropBio tests, and the LE group was defined by the presence of pathology which was classified according to the Dreyer staging protocol (Dreyer et al., 2003). **Table 1** shows total sample size, age, gender, years living in the endemic area, as well as mean rounds of MDA received and median lymphedema stage (where applicable).

NA, not applicable.

### Flow Cytometry of Peripheral Whole Blood Cells

Flow cytometry analysis of peripheral whole blood samples was performed during the survey in Ghana as previously described (Horn et al., 2021). In brief, 100 µl whole blood was taken from a sodium heparin blood collection tube (Sarstedt, Nümbrecht, Germany) which was processed within 8 hours of collection. Whole blood was first incubated at room temperature for 30 min with the following extracellular anti-human antibodies: CD8-V500 (clone RPAT8; BD™ Biosciences, Heidelberg, Germany), CD195 (CCR5)-APC (clone REA245; Miltenyi Biotech, Bergisch Gladbach, Germany), and CD45RA-Bv421 (clone HI100; Biolegend, San Diego, USA). Cells were then lysed for 10 min with 1x BD FACS™ lysis buffer (BD™ Biosciences) and centrifuged (Hettich Rotina 420R, Tuttlingen, Germany) at 600g for 5 min. The cell pellet was then resuspended in a freezing solution (inactivated Fetal Bovine Serum with 10% DMSO) and put into a Nunc™ CryoTube™ (Thermo Fisher Scientific, Waltham, USA) to be frozen at -20°C overnight using a StrataCooler Preservation module (Agilent Technologies, Santa Clara, USA) before long-term storage in liquid nitrogen.

Cells were shipped to Germany, thawed in a 37°C water bath, and washed twice with 2.5 ml of pre-warmed thawing media (RPMI 1640 media GlutaMAX supplement (Invitrogen, Carlsbad, USA) with 10% FCS (Sigma-Aldrich, St. Louis, USA), 1% penicillin-streptomycin (10,000 U/ml, Sigma-Aldrich), and 0.2 µl/ml Benzonase^®^ Nuclease (25 U/µl, Merck Millipore, Kenilworth, USA)). Cells were then additionally washed with PBS and centrifuged at 400g for 5 min before fixation with 1x BD CellFIX (BD™ Biosciences). Cells were then acquired using the CytoFlex S flow cytometer (Beckman Coulter, Brea, USA) and analyzed using FlowJo_v10.6.0 software (FlowJo LLC, Ashland, Oregon, USA). Gating strategy was developed using fluorescence minus one controls, and compensation was performed using VersaComp Antibody Capture Kit (Beckman Coulter).

### Flow Cytometry of Peripheral Blood Mononuclear Cells (PBMCs)

PBMCs were isolated using Leucosep tubes (Sigma-Aldrich), and cells were cryo-preserved in liquid nitrogen during the field survey in Ghana as previously described (Arndts et al., 2012; Arndts et al., 2015). PBMCs were shipped to Germany, thawed, and washed twice with RPMI 1640 medium supplemented with 10% FCS, gentamycin, penicillin/streptomycin (all 50 µg/ml), and L-glutamine (292.3 µg/ml) (Sigma-Aldrich). For antibody staining, cells were permeabilized using the FoxP3 Fixation/Permeabilization kit (Thermo Fisher Scientific) according to the manufacture’s description. Upon permeabilization, cells were incubated for 20 min at 4°C with a 13-colour anti-human antibody panel including CD4-BUV661 (clone SK3), CD8-BUV395 (clone HIT8a), CD39-BV 510 (clone TU66) (all obtained from BD™ Biosciences), IFN-γ-FITC (clone 4S.B3), IL-10-PE (JES3-9D7), T-bet-PE-Cy7 (clone 4B10), Eomes-PE-eFluor 610 (clone WD1928), PD-1-APC-eFluor 780 (clone eBoJ105), LAG-3-eFluor 450 (clone 3DS223H), Tim-3-Super Bright 600 (clone F38-2E2), KLRG-1-PerCP-eFluor 710 (clone 13F12F2), CD127-AF700 (clone eBioRDR5), and TNF-α-APC (clone Mab11). After incubation, cells were washed twice with permeabilization buffer and finally resuspended in 100 μl PBS. Data were acquired on a CytoFlex S flow cytometer (Beckman Coulter) and analyzed using FlowJo_v10.6.0 software (FlowJo LLC). Only samples containing greater than 2000 CD8^+^ T-cell events were included in analysis. The following were removed from analysis: TNF-α (insufficient expression), CD127, and CD4 (CD4 used for accurate gating of CD8^+^ T cells), resulting in n = 10 markers used in the analysis. Unless otherwise stated, all media, supplements, and reagents were purchased from Thermo Fisher Scientific (Life Technologies Corporation, Grand Island, USA). Gating strategy was developed using fluorescence minus one controls, and compensation was performed using VersaComp Antibody Capture Kit (Beckman Coulter).

### Statistical Analysis

Statistical analyses were performed using SPSS software (IBM SPSS Statistics 22; Armonk, NY), CRAN R 3.6.2, and the GraphPad Prism 8.3.0 program (GraphPad Software, Inc., La Jolla, USA). According to the Kolmogorov-Smirnov test, variables showed a non-parametric distribution. Thus, Kruskal-Wallis test was performed for multiple comparison and, if significant, followed by Dunn’s *post hoc* test for a further comparison of the groups. P-values of 0.05 or less were considered significant. For comparisons of continuous parameters in LE and non-LE (EN and Wb-inf.) cohorts, the Spearman correlation was used.

## Results

### Elevated Frequencies of CD8^+^CCR5^+^CD45RA^-^ T Cells in Lymphatic Filariasis Lymphedema Patients

**Figure 1:**
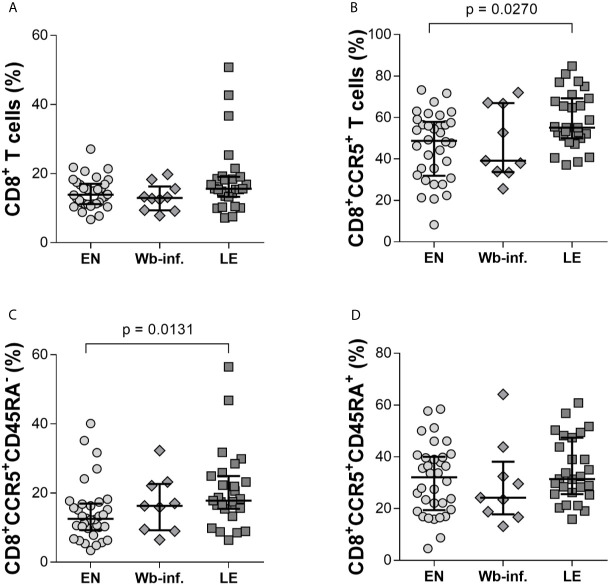
CD8^+^ T cells from individuals with Wuchereria bancrofti-associated lymphedema present elevated expression of CCR5^+^CD45RA^-^. Frequencies of CD8^+^ T cells were measured by flow cytometry in whole blood samples from cohorts of Wuchereria bancrofti infected (Wb-inf., n = 10) and lymphedema (LE) patients (n = 31) and compared with those from endemic normal (EN) subjects (n = 34) living in the same communities **(A)**. Cell populations were analyzed according to the applied gating strategy (**Supplementary Figure S1**). The CD8^+^ T cells were then further analyzed for expression of CCR5 per se **(B)** and in conjunction with memory **(C)**, CD45RA^-^ or naïve **(D)**, CD45RA^+^ markers. Symbols in graphs show individual data sets with median and IQR. Statistical significance between the groups was obtained using Kruskal-Wallis followed by Dunn’s multiple comparison *post hoc* analysis.

Neither pre-clinical nor clinical studies have thoroughly characterized CD8^+^ T cells during *W. bancrofti* infections (Veazey, Acierno et al.). Using multi-parametric flow cytometry, we determined phenotypic and functional characteristics of CD8^+^ T cells in individuals presenting LE as well as EN and asymptomatic *W. bancrofti-*infected cohorts. Using a rapid whole blood staining method (Horn et al., 2021) (**Supplementary Figure S1**), we could quickly assess whether there were differences in the frequencies of activation markers on CD8^+^ T-cell populations, along with other extracellular markers (CD45RA, CCR5), which are associated with memory CD8^+^ T cells (Kohlmeier et al., 2008). While we observed no differences in the proportion of CD8^+^ T cells *per se* between the EN, Wb-infected, and LE cohorts (**Figure 1A**), there were significantly more CD8^+^CCR5^+^ T cells in LE patients (**Figure 1B**) when compared to EN. Further analysis revealed that this increased expression was on memory (CCR5^+^CD45RA^-^) but not naïve (CD45RA^+^) CD8^+^ T cells (**Figures 1C, D**, respectively), suggesting that CD8^+^CCR5^+^CD45RA^-^ T-cell subsets play a role during the development of LE. In summary, it was found that there are significantly higher frequencies of CD8^+^CCR5^+^ T cells and CD8^+^CCR5^+^CD45RA^-^ T cells within the LE cohort.

### Comparable Ratios of CD8^+^ex_mem_ and CD8^+^ex_eff_ T-Cell Subsets Within EN, Wb-Infected, and LE Groups

**Figure 2:**
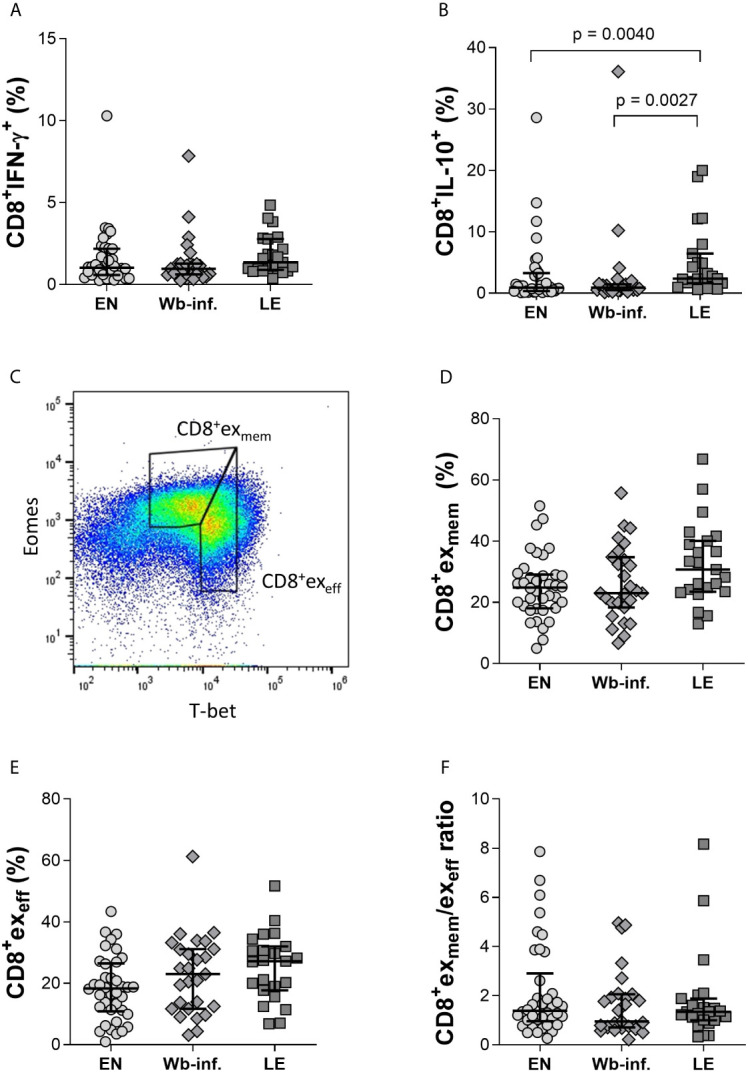
Comparable ratios of CD8^+^ex_mem_ and CD8^+^ex_eff_ T-cell subsets within EN, Wb-infected, and LE groups. Cell populations were analyzed according to the applied gating strategy (**Supplementary Figures S2**, **S3**). Levels of intracellular IFN-γ **(A)** and IL-10 **(B)** from CD8^+^ T cells were measured using isolated PBMC fractions in endemic normal (EN, n = 38), *Wuchereria bancrofti* infected (Wb-inf., n = 27), and lymphedema (LE, n = 23) subjects. Representative gating strategy to distinguish the CD8^+^ex_mem_ (CD8^+^ T-bet^dim^Eomes^hi^) and CD8^+^ex_eff_ (CD8^+^ T-bet^hi^Eomes^dim^) expression on CD8^+^ T cells in PBMC samples **(C)**. The frequencies of CD8^+^ex_mem_ **(D)** and CD8^+^ex_eff_ **(E)** cell subsets in PBMCs were also compared as well as the ratios of CD8^+^ex_mem_/CD8^+^ex_eff_ **(F)**. Symbols in graphs show individual data points with median and IQR. Statistical significances between the groups were obtained using Kruskal-Wallis followed by Dunn’s multiple comparison *post hoc* analysis.

Complementing the analysis performed on cells in whole blood, a 13-colour FACS panel was then designed to explore parameters of exhaustion in CD8^+^ T cells on isolated PBMCs (**Supplementary Figures S2**, **S3**). While similar percentages of CD8^+^IFN-γ^+^T cells were observed between the study groups, frequencies of CD8^+^ T cells expressing IL-10 were significantly increased within the LE group when compared to both EN and Wb-infected groups (**Figure 2A, B**, respectively). Moreover, a positive correlation between the frequency of IL-10^+^- but not IFN-γ^+^-CD8^+^ T cells with LE was observed (r = 0.390 and p < 0.001). Since our flow cytometry results from the whole blood and PBMC samples confirmed previous findings that LE patients are characterized by CD8^+^ T cells expressing IL-10 superfamily member cytokines like IL-10 and IL-26 (Anuradha et al., 2014b), we then characterized CD8^+^ T cells in more detail in regards to effector and memory functions, as well as exhaustion. Thus, PBMCs were stained with an advanced 13-colour flow cytometry panel according to the applied gating strategy (**Supplementary Figure S3**). In brief, whereas T-bet controls the expression of effector functions, Eomes is considered the gatekeeper of the memory CD8^+^ T-cell repertoire (Pauken and Wherry, 2015). Previous studies have shown that comparing expression levels of T-bet and Eomes within CD8^+^ T cells provides information on progenitor and terminally exhausted subsets, respectively (Paley et al., 2012; Buggert et al., 2014; Pauken and Wherry, 2015; Jia et al., 2019). Thus, we compared the frequencies of exhausted memory CD8^+^T-bet^dim^Eomes^hi^ (CD8^+^ex_mem_) and exhausted effector CD8^+^T-bet^hi^Eomes^dim^ (CD8^+^ex_eff_) subpopulations between EN, Wb-infected, and LE groups defined as depicted in **Figure 2C**. No differences were observed between the groups in terms of frequencies of CD8^+^ex_mem_ (**Figure 2D**) or CD8^+^ex_eff_ T-cell subsets (**Figure 2E**). This was also reflected in the ratio of CD8^+^ex_mem_/CD8^+^ex_eff_ (**Figure 2F**). The frequencies of CD8^+^ex_mem_ T cells positively correlated, albeit moderately, with the presence of LE (r = 0.264 and p = 0.0130). Spearman correlation analysis of LE stages with defined cell subsets from PBMC flow cytometry analysis was performed with all 88 individuals (EN, n = 38, Wb-infected, n = 27, LE, n = 23) (**Supplementary Table S1**). This analysis was performed for all of the following correlation analyses with EN and Wb-infected participants listed as stage 0.

### Reduced Levels of IFN-γ in Tim-3^+^ and LAG-3^+^ CD8^+^ex_mem_ Subsets in LE Individuals

**Figure 3:**
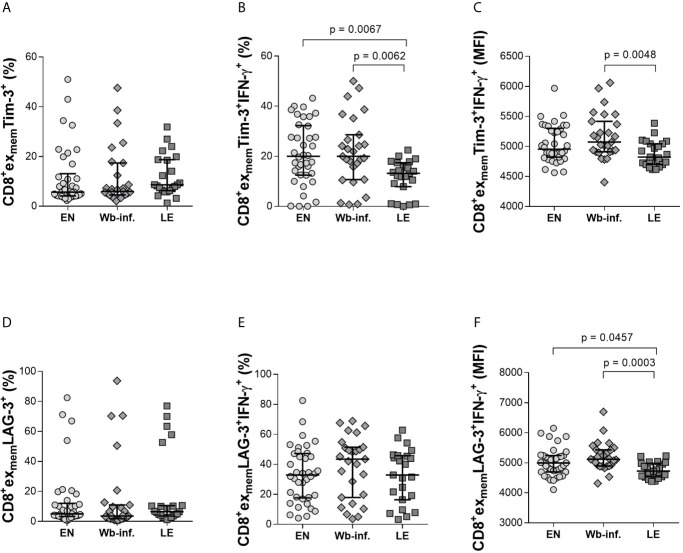
Decreased levels of IFN-γ in CD8^+^ex_mem_ T-cell subsets expressing either Tim-3^+^ and LAG-3^+^ in lymphedema patients. Cell populations were analyzed according to the applied gating strategy (**Supplementary Figure S3**). To determine the exhaustion state of specific immune cells, Tim-3 **(A–C)** and LAG-3 **(D–F)** on CD8^+^ex_mem_ cell subsets were analyzed in PBMC fractions from endemic normals (EN, n = 38), *Wuchereria bancrofti* infected (Wb-inf., n = 27), and participants displaying lymphedema pathology (LE, n = 23). In addition, subsets were analyzed regarding their ability to produce IFN-γ (frequencies [median and IQR] shown in **(B, E)** and median fluorescence intensity (MFI) counts in **(C, F)**. Symbols in graphs show data points with median and IQR. Statistical significances between the groups were obtained using Kruskal-Wallis followed by Dunn’s multiple comparison *post hoc* analysis.

Exhaustion in CD8^+^ T cells transpires from overwhelming antigen load or inflammation which leads to the loss of being able to effectively function. Exhaustion in CD8^+^ T cells is reflected by their increased expression of the cell surface markers PD-1, Tim-3, and elevated IL-10 levels in plasma (Osuch et al., 2020). Thus, we compared expression profiles of CD8^+^ex_mem_ T cells expressing exhaustion markers such as Tim-3, LAG-3, CD39, PD-1, and KLRG-1 with additional IL-10, IFN-γ and TNF-α expression. Whereas the proportion of CD8^+^ex_mem_Tim-3^+^ subsets were comparable between the groups (**Figure 3A**), there was a significant reduction in frequencies of CD8^+^ex_mem_Tim-3^+^IFN-γ^+^cells in the LE group when compared to both EN and Wb-infected cohorts (**Figure 3B**). This was also reflected in median fluorescence intensity (MFI) analysis between Wb-infected and LE individuals indicating a reduced production of IFN-γ by this subset in patients with LE (**Figure 3C**). Furthermore, we found a negative significant correlation with the frequencies of CD8^+^ex_mem_Tim-3^+^ IFN-γ^+^ cells and LE (r = -0.377 and p < 0.001) and also with the MFI of CD8^+^ex_mem_Tim-3^+^ IFN-γ^+^ cells and with LE (r = - 0.323 and p = 0.002). Interestingly, although frequencies of CD8^+^ex_mem_LAG-3^+^ (**Figure 3D**) or CD8^+^ex_mem_LAG-3^+^IFN-γ^+^ (**Figure 3E**) T cells were comparable between the groups, the IFN-γ MFI on LAG-3^+^CD8^+^ex_mem_ T cells of individuals with LE was significantly reduced (**Figure 3F**), strongly indicating a decreased activity in these cells as well, which was further reflected in a significant negative correlation of the MFI and the presence of LE (r = -0.390 and p < 0.001). No significant changes were observed on CD8^+^ex_mem_ populations in other parameters measured, including CD39, KLRG-1, or PD-1 (**Supplementary Table S2**). In regards to TNF-α, expression levels were consistently non-determinable in all analyzed exhausted CD8^+^ T-cell subset, and thus, no conclusion could be drawn from these results.

### Increased Frequencies of IL-10-Expressing CD8^+^ex_eff_ Subsets in LE Individuals Are Associated With CD39, KLRG-1, LAG-3, Tim-3, and PD-1 Expression

**Figure 4:**
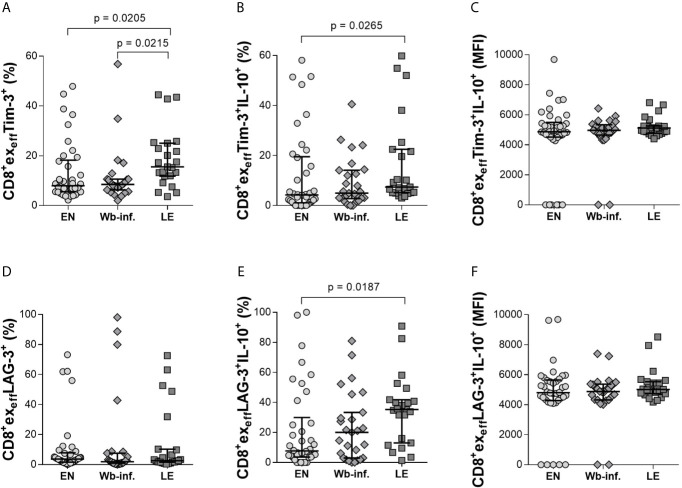
Increased levels of IL-10 in CD8^+^ex_eff_Tim-3^+^ and LAG-3^+^ cell subsets in patients displaying lymphedema. Cell populations were analyzed according to the applied gating strategy (**Supplementary Figure S3**). To determine the exhaustion state of specific immune cells, CD8^+^ex_eff_Tim-3^+^ **(A–C)** and LAG-3^+^ **(D–F)** cell subsets of endemic normals (EN, n = 38), *Wuchereria bancrofti* infected individuals (Wb-inf., n = 27), and participants suffering from lymphedema pathology (LE, n = 23) were analyzed regarding their ability to produce IL-10 [frequencies shown in **(A, B, D, E)** are given in percent, median fluorescence intensity shown in **(C, F)** is given in counts]. Symbols in graphs show data points with median and IQR. Statistical significance between the groups was obtained using Kruskal-Wallis followed by Dunn’s multiple comparison *post hoc* analysis.

**Figure 5:**
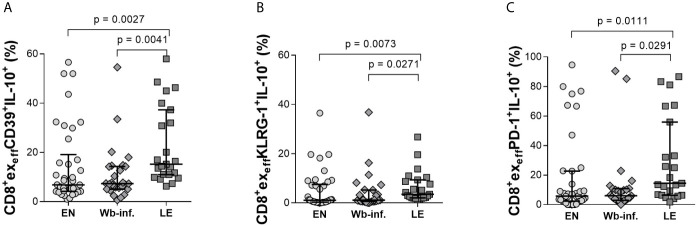
Increased levels of IL-10 in CD39^+^, KLRG-1^+^, and PD-1^+^ CD8^+^ex_eff_ subsets of lymphedema patients. Cell populations were analyzed according to the applied gating strategy (**Supplementary Figure S3**). Next, CD39^+^- **(A)**, KLRG-1^+^- **(B)**, and PD-1^+^- **(C)** CD8^+^ex_eff_ cell subsets isolated from endemic normal (EN, n = 38), *Wuchereria bancrofti* infected, (Wb-inf., n = 27), and participants suffering from lymphedema pathology (LE, n = 23) were analyzed for their levels of IL-10 (frequencies shown) by flow cytometry. Symbols in graphs show each individual value with median and IQR. Statistical significance between the groups was obtained by using Kruskal-Wallis followed by Dunn’s multiple comparison *post hoc* analysis.

Further analysis of effector CD8^+^ T-cell subsets (CD8^+^ex_eff_) also revealed distinct differences in individuals presenting with LE. Again, analyzing Tim-3 on CD8^+^ex_eff_ cells showed elevated frequencies in LE patients when compared to either EN or Wb-infected groups (**Figure 4A**). Differences in CD8^+^ex_eff_Tim-3^+^IL-10^+^ populations were also higher in the LE group when compared to EN cohorts (**Figure 4B**) but not in terms of IL-10 MFI expression on CD8^+^ex_eff_Tim-3^+^ cells (**Figure 4C**). Frequencies of CD8^+^ex_eff_ cells expressing Tim-3 correlated significantly with LE (r = 0.330 and p < 0.001), and additionally, frequencies of CD8^+^ex_eff_Tim-3^+^IL-10^+^ showed a positive correlation with LE (r = 0.269 and p = 0.011). With regards to LAG-3 expression on CD8^+^ex_eff_, no differences were observed in the frequencies of these cells *per se* (**Figure 4D**), but frequencies of IL-10 on this distinct subset were increased in the LE group when compared to EN (**Figure 4E**), which was further highlighted by a positive correlation of these frequencies with LE (r = 0.286 and p = 0.007). However, this was not seen in terms of IL-10 MFI (**Figure 4F**), which reveal the amount of IL-10 secretion from cells, suggesting that the overall frequency of CD8^+^ex_eff_LAG3^+^IL-10^+^ cells was increased in the LE cohort, whereas secretion of IL-10 from single cells remains comparable. Interestingly, this phenotype was not restricted to Tim-3 and LAG-3, since higher frequencies of CD8^+^ex_eff_IL-10^+^ subsets in LE patients were also associated with expression of CD39, KLRG-1, and PD-1 (**Figures 5A–C**, respectively). Moreover, this was also positively correlated to individuals presenting LE. These effects were not observed with CD8^+^ex_mem_IL-10^+^ cells that were expressing either CD39, KLRG-1, or PD-1 (**Supplementary Table S2**). In summary, there was a significantly higher frequency of all the markers examined on the CD8^+^ex_eff_IL-10^+^ subsets within the LE cohort.

## Discussion

Nowadays, it is well known that helminths modulate the host’s immune system in order to ensure both reproduction and long-term survival, and the filarial nematodes are no exception (Aravindhan and Anand, 2017; Moyat et al., 2019; Murthy, 2019; Ayelign et al., 2020). Using advanced flow cytometry, this study has addressed the characteristics of systemic CD8^+^ T-cell populations in different groups of individuals (EN, Wb-infected, and those with LE) living in endemic areas of lymphatic filariasis in the Upper East Region of Ghana. Although CD8^+^ T-cell frequencies revealed no initial differences between the three groups *per se*, further analysis of distinct memory, effector, and exhaustion-related markers revealed a previously unknown immune trait in LE individuals. LE characteristically presents with swelling of the limbs and develops over a substantial period of time. The Dreyer grading system employed in this study provides the means to classify the level of pathology (Dreyer et al., 2003). As stages progress, the skin becomes thick and hard, owing to hyperpigmentation and hyperkeratosis (Zulfiqar and Malik, 2021). There are numerous cytokines, chemokines, and growth factors which have been suggested to drive clearance of worms and development of LE pathology (Debrah et al., 2006; Debrah et al., 2007; Bennuru and Nutman, 2009; Bennuru et al., 2010). For example, several studies have deciphered immune profiles between LE patients and other endemic cohorts focusing on cytokine, chemokine, and CD4^+^ T-cell characteristics and functions. Amongst others, these studies have revealed elevated levels of TNF receptors (Satapathy et al., 2006), and a host of inflammatory markers in the peripheral circulation (C-reactive protein, TNF-α, IL-6, TARC, MCP-1, and IL-10) as reviewed by Babu and Nutman (Babu and Nutman, 2012). A study in nude mice also showed elevated levels of IL-1, IL-6, TNF-α, and GM-CSF in lymph fluid (Rao et al., 1996). In addition, elevated frequencies of Th1, Th9, and Th17 with lower classically helminth-associated Th2 cells were also observed in patients with filarial LE (Babu et al., 2009). Expanding on these earlier findings, we were able to examine a number of extracellular markers to help define CD8^+^ T-cell memory subsets during filariasis; elevated CD8^+^CCR5^+^CD45RA^-^ (memory subset) in LE patients (Veazey et al., 2008). To date, the CCR5 chemokine has not been extensively studied in filariasis, especially in regards to its role on CD8^+^ T cells, although studies do indicate a requirement for CCR5 in early recruitment of memory CD8^+^ T cells to areas of inflammation during respiratory viral infections (Kohlmeier et al., 2008). However, so far, expression patterns of CCR5 on CD8^+^ T-cell populations during helminth infections remain uncertain. CCR5 is commonly known as the most relevant co-receptor for HIV on CD4^+^ T cells (Lederman et al., 2006; Lopalco, 2010). In addition, many viral infections are greatly influenced by CCR5 expression (Ellwanger et al., 2020a), including HIV (Olsson et al., 2000; Fukada et al., 2002; Zaunders et al., 2003). To our knowledge, there have been no studies determining the expression or influence of CCR5 on CD8^+^ T cells within filarial infected individuals, regardless of infection state. One review highlights the association of CCR5 with *Schistosoma* infections, another chronic helminth disease, but could not confirm the classical dogma that lack of CCR5 is associated with less inflammation (Ellwanger et al., 2020b). Our results would suggest the same holds true for filariasis since the LE group had elevated levels of CD8^+^CCR5^+^ T cells in an inflammation setting.

Indications of a potential role of CD8^+^ T cells in driving inflammatory conditions was first noted in LF patients from India, and there, authors noted that IL-10 and TGF-β were not involved in CD8^+^ T-cell regulation (Anuradha et al., 2014a). Increased serum levels of TGF-β have been reported in filarial pathology groups (also shown to drive T-cell exhaustion) which may also play a role in exhausted CD8^+^ T cells in these subsets and should be included in future studies (Anuradha et al., 2014b; Wherry and Kurachi, 2015). Indeed, predominant CD8^+^ infiltrates were already noted in tissue samples of individuals with clinical disease (Freedman et al., 1995). However, filarial-specific T-cell responses expressing IL-26 were linked to symptoms. The association of this IL-10 superfamily cytokine member with LE correlates to our findings here which began with the simple observation that CD8^+^ T cells in LE patients expressed higher amount of IL-10 which also correlated to the presence of LE. This finding then developed into revealing that IL-10 expressing CD8^+^ex_eff_ and the memory cell subset expressing exhaustion markers were significantly increased in the LE cohort. As mentioned earlier, subsets of exhausted CD8^+^ T cells can be distinguished based on their levels of Eomes and T-bet transcription markers and further classified into effector (CD8^+^T-bet^hi^Eomes^dim^) or memory (CD8^+^ T-bet^dim^Eomes^hi^) subsets as described by Buggert and colleagues (Buggert et al., 2014). Indeed, this study revealed that viral specific CD8^+^ T cells, in HIV positive individuals, showed persistent expression of Eomes and inhibitory receptors such as PD-1, even after years of treatment (Buggert et al., 2014). These data supported the idea that inverse expression of T-bet and Eomes is linked to poor viral-responses and indicative that any further strategies to trigger these T cells will still result in insufficient viral clearance (Buggert et al., 2014). We expanded on this analysis further by simultaneously analyzing these subsets with their expression of Tim-3, LAG-3, PD-1, CD39, and KLRG-1 and building a comprehensive picture of exhausted T cells during filariasis. The consequence of exhausted memory populations in LE individuals will require further analysis, and there are no reports that individuals with non-filarial LE have similar immune traits, although it is known that immune functions are significantly lower during secondary LE and that multiple T-cell related networks are upregulated in these conditions (Ogata et al., 2016). In addition, lymphatic dysfunction has been shown to facilitate secondary bacterial and fungal infections and trigger inflammation in the skin and subcutaneous tissue driving the progression of LE (Olszewski et al., 1997; Shenoy et al., 1999; Pfarr et al., 2009) as well as acute dermatolymphangioadenitis. Further analysis needs to be performed to decipher the role of the observed exhausted CD8^+^ T-cell subsets during LE progression and acute dermatolymphangioadenitis, especially since T-cell exhaustion is associated with impaired protective immunity. Indeed, lymphedematous tissues are immunologically vulnerable to infections and neutrophilic dermatosis and immune responses to vaccines have been associated with decreased antibody titers (Sugaya et al., 2012; Yuan et al., 2019); studying these aspects in filarial LE cohorts would reveal the impact of these exhausted cells on such aspects as well.

Moreover, studies have shown more detailed characteristics of exhausted T cells when analyzing multiple exhaustion markers simultaneously. For example, CD8^+^ T-cell exhaustion can be characterized by a number of cell surface markers including PD-1, Tim-3, LAG-3, and IL-10 plasma levels (Osuch et al., 2020), and earlier studies already identified that PD-1 dependent exhaustion of CD8^+^ T cells in malaria facilitated a chronic disease state (Horne-Debets et al., 2013). Multiple marker expression on CD8^+^ T cells is indicative of a more exhausted phenotype than cells expressing singular markers of exhaustion, such as is often the case with LAG-3 and PD-1 (Blackburn et al., 2009; Mishra et al., 2016; Du et al., 2020). Interestingly, regarding the marker Tim-3, co-expression with PD-1 does not necessarily identify a specific subset of exhausted cells (Jones et al., 2008; Golden-Mason et al., 2009; Yi et al., 2010). Another cytokine associated with exhaustion (although not measured in our study) is Cytotoxic T-Lymphocyte-Associated Antigen 4 (CTLA-4), which has been associated with filarial infections (Steel and Nutman, 2003; Babu et al., 2006), and co-expression of CTLA-4 and PD-1 has been shown *in vitro* in *Strongyloides stercoralis* infections to suppress the Th2 response (Rajamanickam et al., 2019). A Th2 response is one of the characteristic features of a filarial infection which helps the infected individual resist infections or development of chronic pathology (Maizels et al., 1993; Rajamanickam et al., 2019). Future studies should analyze the simultaneous expression of multiple exhaustion markers, including CTLA-4, on the same cell and, in addition, assess how profiles change after filarial-specific activation.

In conclusion, this is the first report of CD8^+^ T-cell exhaustion in filarial LE and, moreover, such patterns are correlated with the presence of LE. Although proportionally equivalent, the sample size in each stage (stages 2, 3, and 6) was low, and therefore, correlation analysis was performed with regards to pathology *per se*. T-cell exhaustion occurs when persistent antigen loads and inflammation lead cells to become exhausted, thus impairing their effector functions (Buggert et al., 2014). Interestingly, the LE cohort studied here had distinct effector and memory exhaustion profiles, including reduced frequency expression of IFN-γ in Tim-3^+^ and LAG-3^+^ CD8^+^ex_mem_ subsets in LE individuals, with enhanced IL-10 expressing CD8^+^ex_eff_ subsets. Indeed, these latter populations occurred with the majority of the tested associated exhaustion markers (Tim-3, LAG-3, CD39, PD-1, and KLRG-1). LE patients usually clear the infection, and indeed, in our PBMC cohort, no antigen positive individuals were observed. Since healthy endemic normal and *W. bancrofti*-infected individuals do not show increased exhausted CD8+ T-cell populations, we suggest that the observed exhaustion phenotype is not driven by circulating filarial antigens. However, LE individuals suffer from secondary infections which are often followed by acute dermatolymphangioadenitis attacks (ADLA) that drive LE progression and maybe exhaustion of CD8^+^ T-cell subsets. To determine if severity and chronicity of LE drives exhaustion, a larger cohort of individuals presenting each LE stage would be required since sample numbers in this study were too low to make an overall conclusion (**Supplementary Table S1**). Future studies and clinical trials should increase the number and spectrum of LE participants, gather more information about secondary infections and ADLA, as well as take these varying exhaustion patterns into consideration for prevention and control management of filarial LE. In addition, the role of exhausted CD4^+^ T cells should also be investigated to broaden our knowledge about the complex interplay of different cell populations during filarial diseases and development of pathology.

## Data Availability Statement

The original contributions presented in the study are included in the article/**Supplementary Material**. Further inquiries can be directed to the corresponding authors.

## Ethics Statement

The studies involving human participants were reviewed and approved by the Committee on Human Research Publication and Ethics at the University of Science and Technology in Kumasi (CHRPE/AP/144/20), the Ethics Committee at the University Hospital of Bonn, Germany (Lfd. 041/18), and the Ethics Committee of the LMU Munich, Germany (17-858 and 18-377). The patients/participants provided their written informed consent to participate in this study.

## Author Contributions

LD, AD, MC, IK, and LL conceived and designed the study. SH, DB-W, AW, IK, LD, AD, JO-M, and KA organized field studies and acquired samples. SH, DB-W, AW, MR, KA, and LL processed samples, performed analysis, and interpreted the data. SH, DB-W, IK, MR, KA, and LL drafted the manuscript, while LD, AD, JO-M, AW, MC, and AH critically revised the article and controlled the intellectual content. All authors contributed to the article and approved the submitted version.

## Funding

This work was supported by the German Research Foundation (Deutsche Forschungsgemeinschaft – DFG) (RHINO project) [grants KR3615/1-1 and HO2009/11-1], German Federal Ministry of Education and Research (Bundesministerium für Bildung und Forschung - BMBF) [grant 01KA1601], and the German Center for Infection Research (Deutsches Zentrum für Infektionsforschung – DZIF) [grant TI03.907_00].

## Conflict of Interest

The authors declare that the research was conducted in the absence of any commercial or financial relationships that could be construed as a potential conflict of interest.

## Publisher’s Note

All claims expressed in this article are solely those of the authors and do not necessarily represent those of their affiliated organizations, or those of the publisher, the editors and the reviewers. Any product that may be evaluated in this article, or claim that may be made by its manufacturer, is not guaranteed or endorsed by the publisher.
