# Supplementary material for: Distinct Immune Profiles of Exhausted Effector and Memory CD8+ T Cells in Individuals With Filarial Lymphedema
Source: Front Cell Infect Microbiol. 2021 Aug 11;11:680832. doi: 10.3389/fcimb.2021.680832 (PMC8415778; doi:10.3389/fcimb.2021.680832)
Supplement: Supplementary file 1 [file Presentation_1.pptx]

## Slide 1
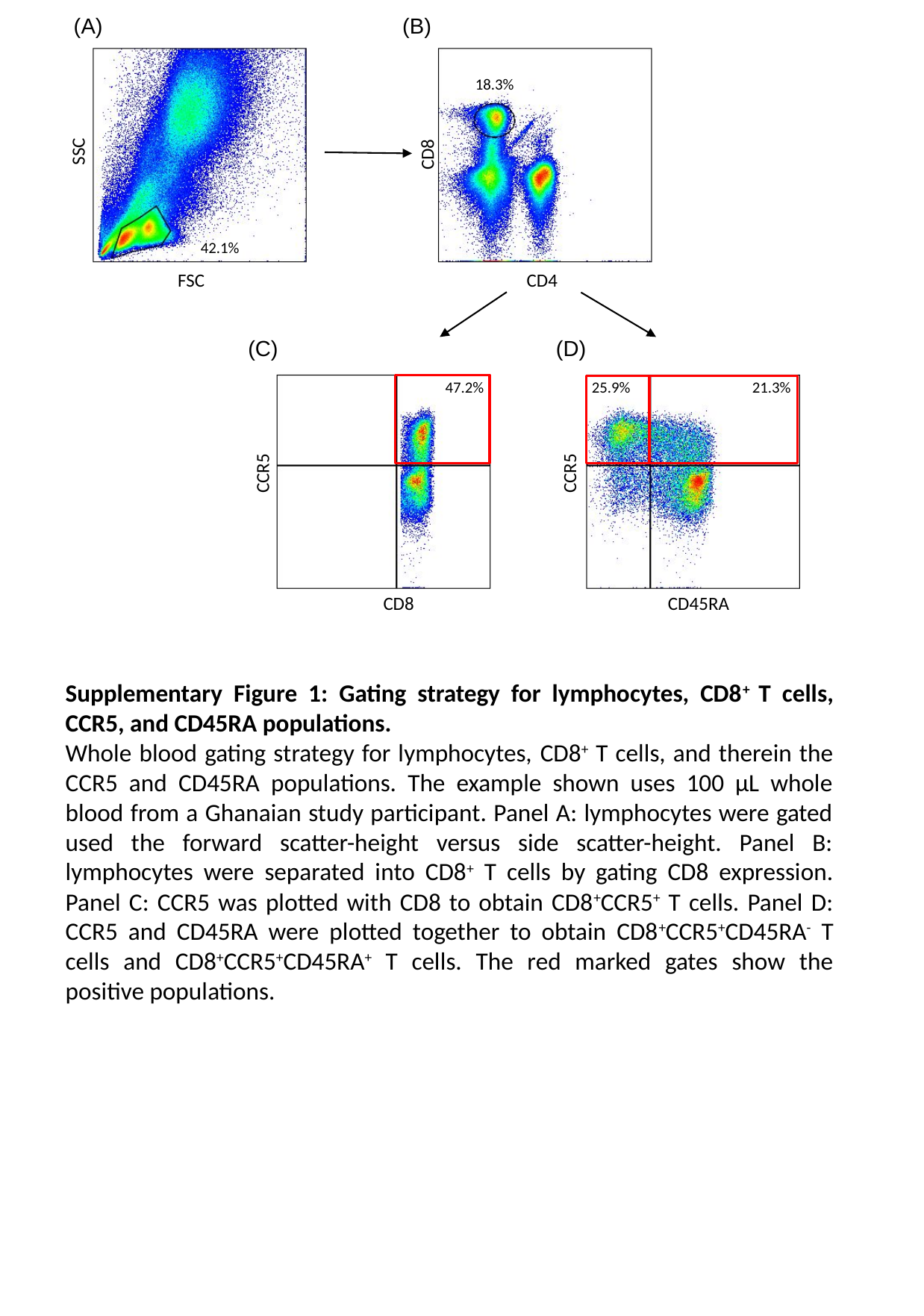

(A)
(B)
18.3%
SSC
CD8
42.1%
FSC
CD4
(C)
(D)
47.2%
25.9%
21.3%
CCR5
CCR5
CD8
CD45RA
Supplementary Figure 1: Gating strategy for lymphocytes, CD8+ T cells, CCR5, and CD45RA populations.
Whole blood gating strategy for lymphocytes, CD8+ T cells, and therein the CCR5 and CD45RA populations. The example shown uses 100 µL whole blood from a Ghanaian study participant. Panel A: lymphocytes were gated used the forward scatter-height versus side scatter-height. Panel B: lymphocytes were separated into CD8+ T cells by gating CD8 expression. Panel C: CCR5 was plotted with CD8 to obtain CD8+CCR5+ T cells. Panel D: CCR5 and CD45RA were plotted together to obtain CD8+CCR5+CD45RA- T cells and CD8+CCR5+CD45RA+ T cells. The red marked gates show the positive populations.

## Slide 2
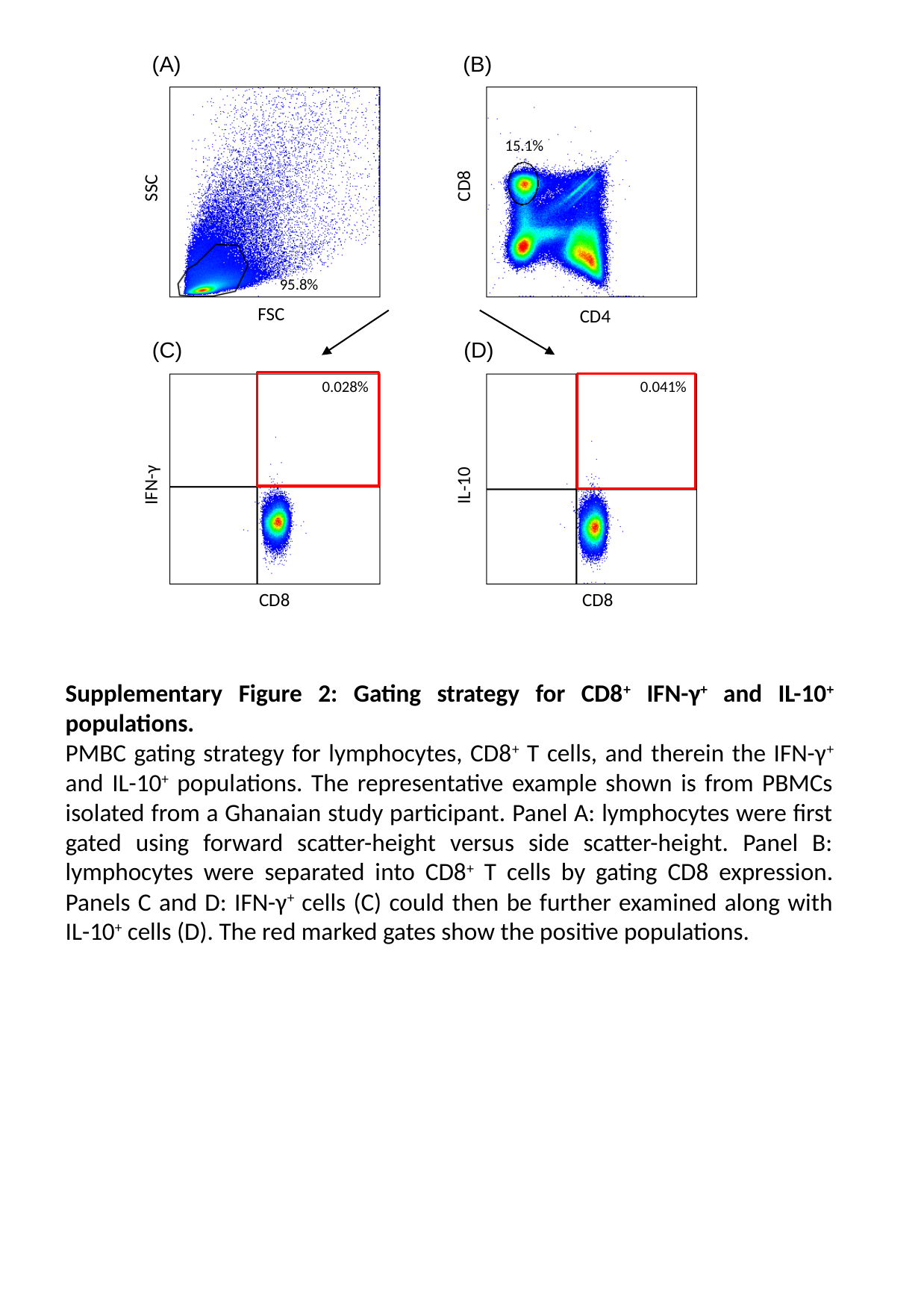

(A)
(B)
15.1%
CD8
SSC
95.8%
FSC
CD4
(C)
(D)
0.041%
0.028%
IL-10
IFN-γ
CD8
CD8
Supplementary Figure 2: Gating strategy for CD8+ IFN-γ+ and IL-10+ populations.
PMBC gating strategy for lymphocytes, CD8+ T cells, and therein the IFN-γ+ and IL-10+ populations. The representative example shown is from PBMCs isolated from a Ghanaian study participant. Panel A: lymphocytes were first gated using forward scatter-height versus side scatter-height. Panel B: lymphocytes were separated into CD8+ T cells by gating CD8 expression. Panels C and D: IFN-γ+ cells (C) could then be further examined along with IL-10+ cells (D). The red marked gates show the positive populations.

## Slide 3
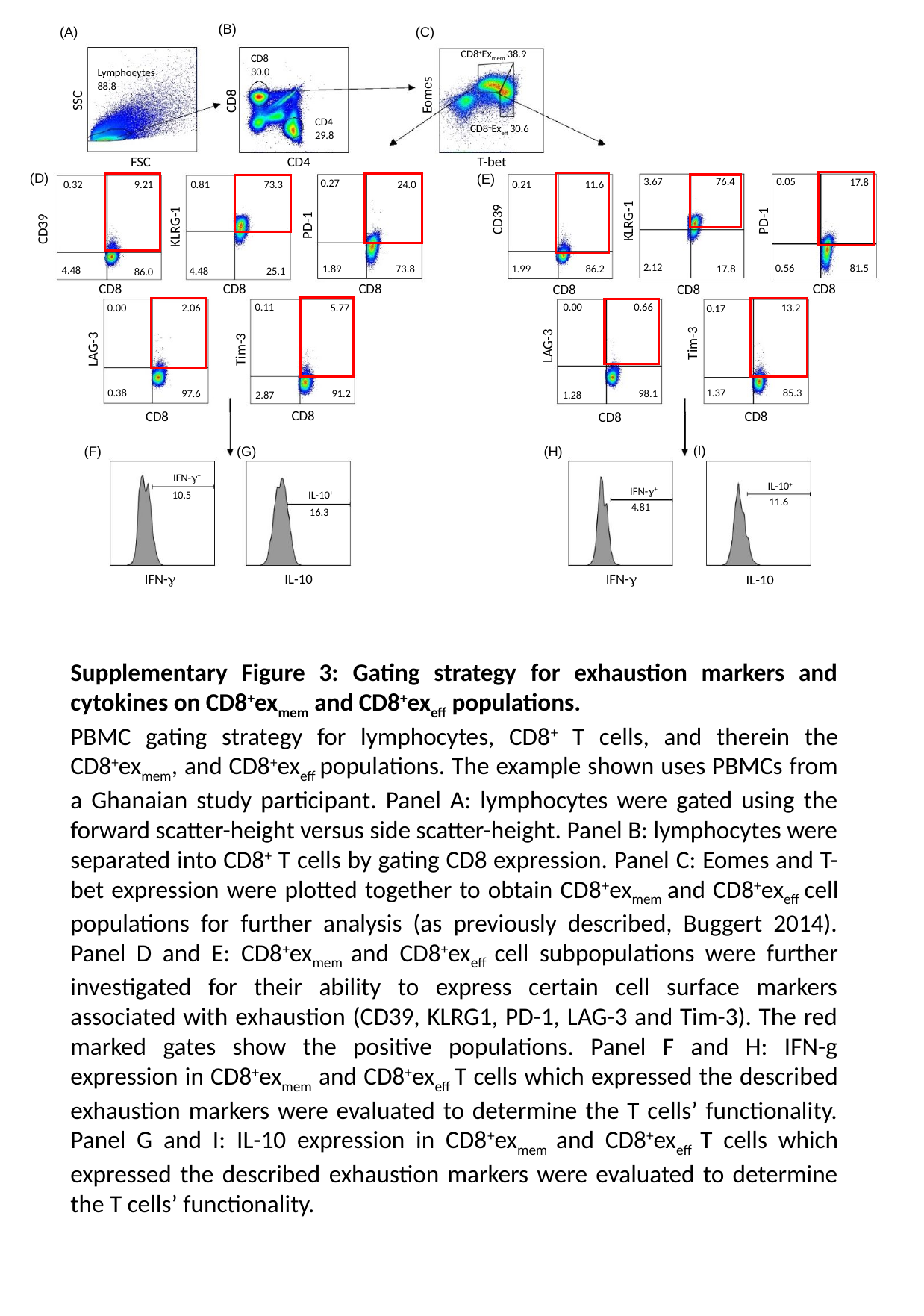

(B)
(C)
(A)
CD8+Exmem 38.9
CD8
30.0
Lymphocytes
88.8
Eomes
SSC
CD8
CD4
29.8
CD8+Exeff 30.6
FSC
CD4
T-bet
(D)
(E)
0.05
76.4
3.67
17.8
0.27
0.21
11.6
0.32
0.81
24.0
73.3
9.21
CD39
PD-1
PD-1
KLRG-1
CD39
KLRG-1
2.12
0.56
81.5
86.2
17.8
1.99
73.8
1.89
4.48
4.48
25.1
86.0
CD8
CD8
CD8
CD8
CD8
CD8
0.11
0.00
0.66
5.77
13.2
2.06
0.00
0.17
Tim-3
LAG-3
Tim-3
LAG-3
0.38
1.37
85.3
97.6
91.2
98.1
1.28
2.87
CD8
CD8
CD8
CD8
(I)
(F)
(G)
(H)
IFN-g+
IL-10+
IFN-g+
IL-10+
10.5
11.6
4.81
16.3
IFN-g
IFN-g
IL-10
IL-10
Supplementary Figure 3: Gating strategy for exhaustion markers and cytokines on CD8+exmem and CD8+exeff populations.
PBMC gating strategy for lymphocytes, CD8+ T cells, and therein the CD8+exmem, and CD8+exeff populations. The example shown uses PBMCs from a Ghanaian study participant. Panel A: lymphocytes were gated using the forward scatter-height versus side scatter-height. Panel B: lymphocytes were separated into CD8+ T cells by gating CD8 expression. Panel C: Eomes and T-bet expression were plotted together to obtain CD8+exmem and CD8+exeff cell populations for further analysis (as previously described, Buggert 2014). Panel D and E: CD8+exmem and CD8+exeff cell subpopulations were further investigated for their ability to express certain cell surface markers associated with exhaustion (CD39, KLRG1, PD-1, LAG-3 and Tim-3). The red marked gates show the positive populations. Panel F and H: IFN-g expression in CD8+exmem and CD8+exeff T cells which expressed the described exhaustion markers were evaluated to determine the T cells’ functionality. Panel G and I: IL-10 expression in CD8+exmem and CD8+exeff T cells which expressed the described exhaustion markers were evaluated to determine the T cells’ functionality.
